# Supplementary material for: Identification of photoperiod-induced specific miRNAs in the adrenal glands of Sunite sheep (Ovis aries)
Source: Front Vet Sci. 2022 Jul 22;9:888207. doi: 10.3389/fvets.2022.888207 (PMC9354845; doi:10.3389/fvets.2022.888207)
Supplement: Supplementary file 1 [file Data_Sheet_1.ZIP › Supplementary file/miRDeep2 file.zip!/miRDeep2 file/Novel_Structure/LP42A3/Novel_22.pdf]

Provisional ID	:	NC_019484.2_24413
Score total	:	2
Score for star read(s)	:	-1.3
Score for read counts	:	-0.3
Score for mfe	:	2.1
Score for randfold	:	1.6
Score for cons. seed	:	
Total read count	:	11
Mature read count	:	11
Loop read count	:	0
Star read count	:	0

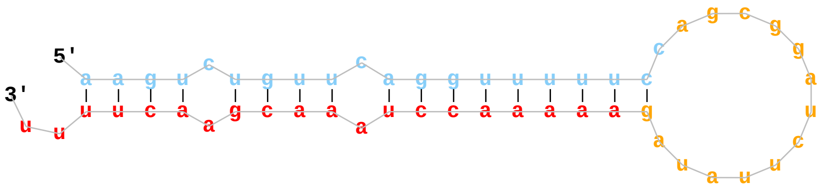

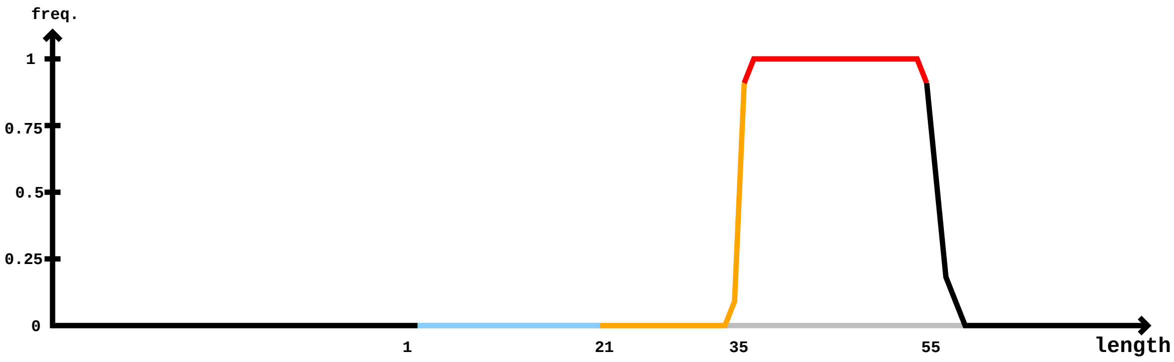

Star Mature

```
5'- cacucacaacccaaauguaauauuggguuggccaaaaagucuguucagguuuuuccagcggaucuuauagaaaaaccuaaacgaacuuuuuggccaacccaauacuugguuc   -3'    exp
    ..........((((..(((...((((((((((((((((((.((((.(((((((((..............))))))))).)))).)))))))))))))))))))))))))...    reads       mm          sample
    .....................................................................gaaaaaccuGaacgaacuuuu......................    1           1           seq
    ......................................................................aaaaaccuGaacgaacuuu.......................    1           1           seq
    ......................................................................aaaaaccuGaacgaacuuuu......................    7           1           seq
    ......................................................................aaaaaccuaaacgaacuuuuug....................    1           0           seq
    .......................................................................aaaaccuGaacgaacuuuuugg...................    1           1           seq
```
